# Optical tomographic imaging of near infrared imaging agents quantifies disease severity and immunomodulation of experimental autoimmune encephalomyelitis in vivo

**DOI:** 10.1186/1742-2094-10-138

**Published:** 2013-11-15

**Authors:** Valerie L Eaton, Kristine O Vasquez, Gwendolyn E Goings, Zoe N Hunter, Jeffrey D Peterson, Stephen D Miller

**Affiliations:** 1 Department of Microbiology-Immunology, Northwestern University, Feinberg School of Medicine, 6-713 Tarry Building, 303 E Chicago Avenue, Chicago, IL 60611, USA; 2 Applied Biology & In Vivo Discovery Services, PerkinElmer, Inc, 68 Elm St, Hopkinton, Boston, MA 01748, USA

**Keywords:** Experimental autoimmune encephalomyelitis (EAE), Protease, Molecular imaging, Near infrared fluorescence

## Abstract

**Background:**

Experimental autoimmune encephalomyelitis (EAE) is an animal model that captures many of the hallmarks of human multiple sclerosis (MS), including blood–brain barrier (BBB) breakdown, inflammation, demyelination and axonal destruction. The standard clinical score measurement of disease severity and progression assesses functional changes in animal mobility; however, it does not offer information regarding the underlying pathophysiology of the disease in real time. The purpose of this study was to apply a novel optical imaging technique that offers the advantage of rapid imaging of relevant biomarkers in live animals.

**Methods:**

Advances in non-invasive fluorescence molecular tomographic (FMT) imaging, in combination with a variety of biological imaging agents, offer a unique, sensitive and quantifiable approach to assessing disease biology in living animals. Using vascular (AngioSense 750EX) and protease-activatable cathepsin B (Cat B 680 FAST) near infrared (NIR) fluorescence imaging agents to detect BBB breakdown and inflammation, respectively, we quantified brain and spinal cord changes in mice with relapsing-remitting PLP_139-151_-induced EAE and in response to tolerogenic therapy.

**Results:**

FMT imaging and analysis techniques were carefully characterized and non-invasive imaging results corroborated by both *ex vivo* tissue imaging and comparison to clinical score results and histopathological analysis of CNS tissue. FMT imaging showed clear differences between control and diseased mice, and immune tolerance induction by antigen-coupled PLGA nanoparticles effectively blocked both disease induction and accumulation of imaging agents in the brain and spinal cord.

**Conclusions:**

Cat B 680 FAST and AngioSense 750EX offered the combination best able to detect disease in both the brain and spinal cord, as well as the downregulation of disease by antigen-specific tolerance. Non-invasive optical tomographic imaging thus offers a unique approach to monitoring neuroinflammatory disease and therapeutic intervention in living mice with EAE.

## Background

The experimental autoimmune encephalomyelitis (EAE) model of MS captures many of the hallmarks of the human disease [1,2]. EAE can be induced in specific strains of mice by immunization with CNS tissue homogenates, purified myelin proteins or peptides derived from these proteins, providing a valuable model for the assessment of the immune-related cellular and molecular contributors to disease induction or progression [3,4].

EAE is generally assessed using a subjective clinical score assessment, which does not provide information about underlying cellular or molecular processes and does not always reflect the underlying pathological changes [5]. Additional information relative to the understanding of disease can be obtained through the study of excised CNS tissues, with standard histology approaches [6], novel approaches in multiplex staining [7], confocal microscopy [8] and label-free spectroscopy [9]. A variety of clinical imaging modalities have been applied to EAE studies, including positron emission tomography (PET), single-photon emission computed tomography (SPECT) and magnetic resonance imaging (MRI). PET and SPECT imaging, although useful in EAE [10,11], are of limited broad utility because of the expense of the imaging systems and the challenges of working with radioactive tracers. MRI imaging is also expensive and can show a poor correlation between MRI findings and clinical symptoms [12], although advances in targeted MRI contrast agents [13,14] are beginning to provide more useful biological and immunological information.

Optical imaging is a relatively new modality and offers the potential advantage of rapid imaging of biological markers. Approaches with bioluminescence and fluorescence in demyelination research have relied on either transgenic luciferase-expressing mice [15] or systemically injected fluorescence imaging agents [16], respectively, relying on 2D surface bioluminescence or epifluorescence signals. More recent advances in fluorescence molecular tomography (FMT) offer a simple and inexpensive imaging option for preclinical research with the benefits of deep tissue detection and improved accuracy of quantification [17-19]. Here, for the first time we apply FMT in conjunction with near infrared (NIR) agents to test the hypothesis that we can detect demyelinating disease and therapeutic efficacy in the brain and spinal cord of EAE mice. A variety of NIR agents were useful in detecting changes associated with disease, including protease activatable agents to detect cathepsin and matrix metalloprotease (MMP) activity associated with inflammation (Cat B 680 FAST^TM^, ProSense® 750EX and MMPSense® 750 FAST), enzymatic renin activity associated with abnormal renin-angiotensin-system activity in the CNS (ReninSense 680 FAST^TM^) and vascular agents designed to detect blood–brain barrier breakdown (AngioSense® 680, AngioSense® 750EX). Imaging and analysis techniques were carefully characterized and non-invasive imaging results corroborated by both *ex vivo* tissue imaging and comparison to clinical score results. Cat B 680 FAST and AngioSense 750EX offered a combination best able to detect disease in the brain and spinal cord, as well as the downregulation of disease by antigen-specific tolerance.

## Materials and methods

### Induction and clinical evaluation of EAE

For the PLP_139-151_-induced experimental autoimmune encephalomyelitis experiments, specific pathogen-free female SJL/J mice (6 to 8 weeks of age) were purchased from Harlan Laboratories (Indianapolis, IN) and housed at the Center for Comparative Medicine at Northwestern University (Chicago, IL) under a controlled environment (72°F; 12:12-h light–dark cycle) under specific pathogen-free conditions with water and food provided *ad libitum*. All experiments were performed in accordance with Northwestern University IACUC guidelines for animal care and use. Proteolipid protein (PLP)_139–151_ (HSLGKWLGHPDKF) and ovalbumin (OVA)_323–339_ (ISQAVHAAHAEINEAGR) were purchased from Genemed Synthesis. EAE was elicited by immunization with 50 μg of PLP_139-151_ in complete Freund’s adjuvant (CFA) supplemented with 200 μg heat-killed *M. tuberculosis* H37Ra (Difco Laboratories, Detroit, MI). A volume of 0.1 ml of emulsion was distributed subcutaneously over three spots on the dorsal flanks on day 0. Observational clinical scores for each mouse were recorded daily using a scale of 0–5 as listed below:

Clinical Score of 0: No abnormalities

Clinical Score of 1: Limp tail or hind limb weakness

Clinical Score of 2: Both limp tail and hind limb weakness

Clinical Score of 3: Partial hind limb paralysis

Clinical Score of 4: Total hind limb paralysis

Clinical Score of 5: Moribund

### Tolerance induction with Ag-coupled nanoparticles

As previously described [20], 500-nm carboxylated PLGA microparticles were purchased from Phosphorex, Inc. (Fall River, MA), and peptide antigens were attached using ECDI (1-ethyl-3-(3′-dimethylaminopropyl)carbodiimide; EMD Chemicals Inc., Gibbstown, NJ) with 0.08 mg of peptide in the presence of 0.32 mg ECDI per 1.0 mg of PLG nanoparticles. Animals received intravenous injections of approximately 9 × 10^9^ nanoparticles comprising 10–15 μg of peptide, depending on the peptide sequence used in the coupling reaction.

### Fluorescent agents for the detection of inflammation

Six commercially available imaging agents (PerkinElmer Inc., Waltham, MA) were used to optimize EAE imaging and detect therapeutic efficacy (Table 1). AngioSense is a vascular imaging agent; ProSense and Cat B detect regions of increased lysosomal cathepsin activity (ProSense is a pan cathepsin agent, while Cat B is preferentially cleaved by cathepsin B); ReninSense FAST is activated by kidney renin.

**Table 1:** Characteristics of fluorescent imaging agents

**Agent**	**Description**	**M.W.**	**Peak Ex/Em**	**Biological activity**
Cat B 680 FAST	Cathepsin-activatable	33 kDa	675/693	Macrophage/microglia
MMPSense 750 FAST	Metalloproteinase-activatable	43 kDa	749/775	Macrophage/microglia
ProSense 750 EX	Cathepsin-activatable	450 kDa	750/770	Macrophage/microglia
ReninSense 680 FAST	Renin-activatable	43 kDa	675/693	T cell RAS activity
AngioSense 680	Vascular agent	250 kDa	680/700	BBB breakdown
AngioSense 750 EX	Vascular agent	70 kDa	750/770	BBB breakdown

### In vivo fluorescence imaging

**Figure 1:**
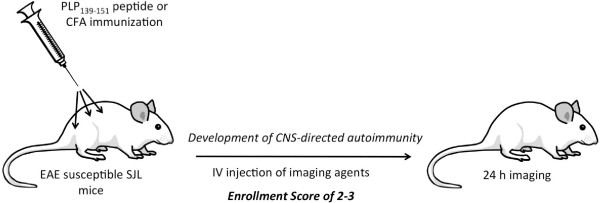
Experimental autoimmune encephalomyelitis induction and imaging **Experimental autoimmune encephalomyelitis induction and imaging.** SJL/J mice were immunized with PLP_139-151_ in CFA as described in the Materials and Methods section. Mice were monitored for clinical signs of disease and enrolled for imaging agent injection upon achieving a score of 2 or 3, and asymptomatic mice were excluded from imaging. Control mice (immunized with CFA only) were injected with imaging agents on day 14. All mice were imaged 24 h after imaging agent injection. For treatment studies, all mice (including asymptomatic mice from the positive control group) were injected on day 13 for imaging on day 14. Two separate FMT tomographic scans (head and spine) were performed, and excised brains and spinal cords were imaged by FMT epifluorescence to confirm *in vivo* results.

Mice were maintained on a low fluorescence alfalfa-free diet (Harlan 2019) recommended for fluorescence imaging, and imaging was performed at the peak of disease (d15). The mice were injected intravenously with 2× the recommended dose of fluorogenic agents (to facilitate brain biodistribution) at d14 and imaged 24 h later (Figure 1). Prior to the imaging, the animals were anesthetized with an i.p. injection of ketamine (100 mg/kg) and xylazine (20 mg/kg) in PBS, and hair from each mouse was removed using depilatory cream (Nair, Church & Dwight Co., Ewing, NJ). The cream was applied 2–3 times, for only 4–5 min each time, to minimize harm to the skin. The animals were then imaged using the FMT 4000™ fluorescence tomography *in vivo* imaging system (PerkinElmer, Hopkinton, MA). For whole body imaging, the anesthetized mouse was placed in a prone position on the imaging cassette to capture the spine or brain of the animal within the imaging scan field. The imaging cassette was adjusted to the proper depth to gently restrain each mouse and then inserted into the heated docking system (regulated at ~37°C) in the FMT imaging chamber. For imaging the head, the mouse was positioned prone on a resin block (designed to mimic optical scattering and absorption properties of the mouse’s body and minimize imaging artifacts from air-containing spaces in the head, such as ear canals and sinuses) and positioned in the imaging cassette. A NIR laser diode was used to transilluminate (*i.e.,* pass light through the body of the animal to be collected on the opposite side) each mouse’s body, with signal detection occurring via a thermoelectrically cooled CCD camera placed on the opposite side of the imaged animal. Appropriate optical filters allowed collection of both fluorescence and excitation data sets, and the multiple source-detector fluorescence projections were normalized to the paired collection of laser excitation data. The entire image acquisition sequence took approximately 6–8 min per mouse. FMT 4000 epifluorescence imaging was routinely performed prior to each tomographic imaging session using built-in LED front illuminators and collection of single camera images.

Animals that were imaged *in vivo* were then euthanized by exsanguination following anesthesia. The mice were transcardially perfused with cold PBS, and the brains and spinal cords were removed post-mortem then imaged by epifluorescence imaging using the FMT 4000.

### FMT reconstruction and analysis

The collected fluorescence data sets were reconstructed by FMT 4000 system software (TruQuant^TM^, PerkinElmer, Waltham MA) for the quantification of fluorescence signal within the head and spine. Three-dimensional regions of interest (ROI) were drawn to encompass each region, and a modest threshold was applied identically to all animals within each agent group in order to minimize background signal. Briefly, the brain ROI for each mouse was placed as a 9 × 12 × 6-mm (w × h × d) ellipsoid in the appropriate head region, and a threshold was applied equal to 30% of the average maximum fluorescence (in nM) of the control brain ROIs. For spinal cord ROIs, each was placed as a 6 × 31 × 4-mm rectangular prism with thresholding applied as 10% of the average maximum fluorescence (nM) of the control spinal cord ROIs. The total amount of brain and spinal cord fluorescence (in pmoles) was automatically calculated relative to internal standards generated with known concentrations of the appropriate NIR dyes. For visualization and analysis purposes, TrueQuant software provided 3D images and tomographic slices.

### Statistical analysis

Comparisons of the mean peak disease severity between any two groups of mice were analyzed by Student’s *t* test. *P* values less than 0.05 were considered significant.

## Results

### Imaging CNS vascular leak in EAE with AngioSense 750EX

To assess whether mouse EAE can be effectively imaged by FMT, we used an NIR vascular imaging agent, AngioSense 750EX (AS750), to detect vascular leaks in the blood–brain barrier associated with CNS disease. On day zero, SJL/J mice were immunized with PLP_139-151_ in complete Freund’s adjuvant (CFA) as described in the Materials and Methods section. Injection sites (3 per mouse) were placed laterally on the backs of the mice to avoid interference with spinal cord imaging. PLP peptide-immunized mice achieved mild disease (average clinical score of 1.4) with 60% disease incidence. Mice were injected with 4 nmol of AS750 when disease severity in half of the mice achieved a clinical score of 2–3 (day 14), and imaging was performed 24 h later to allow optimal accumulation of the agent in BBB-compromised areas of the CNS.

**Figure 2:**
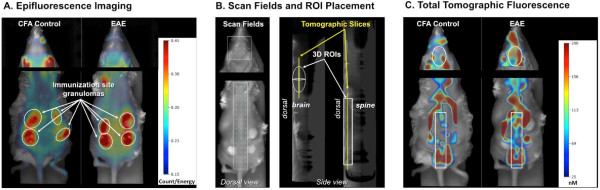
**EAE image acquisition and analysis of AS750. (A)** Whole-body epifluorescence AS750 signal was acquired to show signal within the sites of immunization. **(B)** FMT tomographic scan fields (*left panel*) show the transillumination scan pattern performed for the spine and head regions. 3D ROI placement and sizing is shown for the separate head and spine scans (*right panel*). **(C)** Total fluorescent signal in 3D throughout the spine and head regions is shown to reveal the complexity of bodily signal following AS750 injection.

FMT epifluorescence imaging (Figure 2A) shows that AS750 accumulated as expected within the immunization sites in both a representative CFA-immunized control and a PLP_139-151_-immunized mouse, revealing vascular leak within the resulting granulomas. Transillumination scan fields (required for FMT tomographic imaging) were positioned based upon the epifluorescence images in order to capture the head and spinal cord in two separate 2–4-min scans (Figure 2B). Tomographic fluorescence data sets were acquired, reconstructed in 3D by the FMT software and represented as 3D image overlays on the mouse image. Regions of interest (ROI) were placed to quantify brain (ellipsoidal ROI) and lumbar/sacral spinal cord (rectangular prism) fluorescent signal in three dimensions, and the resulting tomographic fluorescent images are shown in Figure 2C.

**Figure 3:**
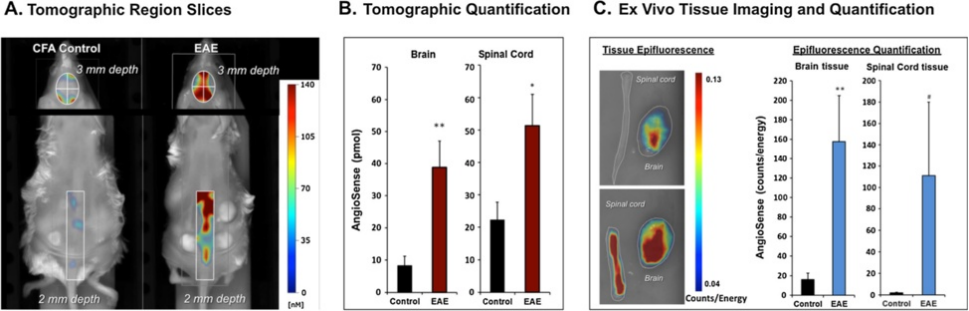
**Quantifying BBB disruption.** PLP_139-151_-immunized mice were injected intravenously with AS750 upon achieving a clinical score of 2 or 3 and imaged by FMT 4000 using tomographic scanning capabilities. CFA control mice were injected 14 days following immunization. **(A)** NIR tomographic fluorescent signal in 2–3-mm slices through the spinal cord and brain regions of representative control and diseased mice. **(B)** NIR tomographic quantification of the fluorescent signal (mean pmol ± SE) in the brains and spinal cord of all experimental mice. **(C)** Brains and spinal cord tissues of the same representative mice shown in **A** were imaged by epifluorescence on the FMT (*left panel*). Epifluorescence quantification (*right panel*) for tissues from all mice. Study is representative of three separate experiments, #*p* < 0.05, *p < 0.01, **p < 0.005).

To better reveal the differences in AS750 localization within the specific anatomical regions in which disease occurs, appropriate tomographic slices were made through the ROIs of the spine (2 mm depth) and brain (3 mm depth). The resulting slice images (Figure 3A) reveal a dramatic difference between the CFA control and the EAE mouse by excluding extraneous non-CNS signal. Quantification of the entire 3D ROI data sets (i.e., all slices per ROI) from the brain and spine of all of the mice (Figure 3B) was performed with modest thresholding applied to minimize background signal in the control mice (described in Materials and Methods). Quantification revealed highly significant elevations in fluorescent signal associated with EAE. Interestingly, although disease incidence (as defined by clinical score) was 60%, all of the EAE mice, including those that were asymptomatic (i.e., no hindlimb weakness or gait abnormalities), showed elevated brain fluorescence (*i.e.,* fluorescence greater than control mean + 2 SD), and 60% showed elevated spine signal. These non-invasive imaging results were confirmed by *ex vivo* epifluorescence imaging and quantification of excised brain and spinal cord tissue (Figure 3C), with both the images and epifluorescence measurements in strong agreement with non-invasive *in vivo* imaging.

### Imaging changes in CNS protease activity in EAE mice

Upregulated proteolytic activity is associated with a number of diseases, including cancer, inflammation, neurodegeneration, cardiovascular disease, arthritis, and diabetes [21-23]. In MS, both matrix metalloproteases (MMP) [24] and cathepsins [25,26] are expressed in inflammatory cells and have been implicated as playing a role in BBB breakdown and/or direct myelin destruction. Even renin, a protease generally associated with regulation of blood pressure, has been implicated in the regulation of inflammatory cell influx [27], supporting a proposed role for the tissue-specific renin-angiotensin system in MS pathogenesis.

**Figure 4:**
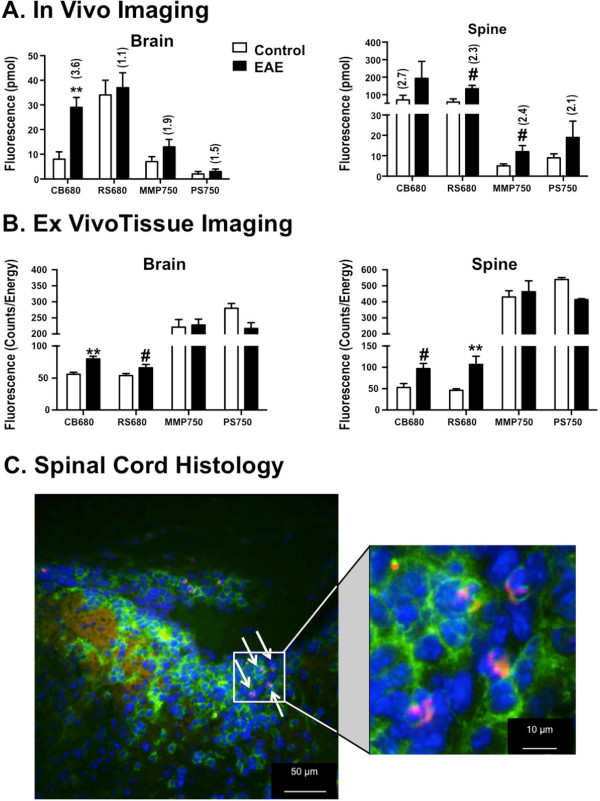
**Imaging protease activity *in vivo*.** PLP_139-151_-immunized mice were injected intravenously with various protease-activatable NIR fluorescence imaging agents [CB680 for cathepsin B activity; PS750 for pan-cathepsin activity (Cat B, L, S, K, V); MMP750 for pan-matrix metalloprotease activity (MMPs 2, 7, 9, 12, 13 and others); and RS680 for renin activity] upon achieving a clinical score of 2 or 3 and imaged by FMT 4000 using tomographic scanning capabilities. CFA control mice were injected 14 days following immunization. **(A)** Quantification of *in vivo* tomographic fluorescence data sets. **(B)** Quantification of epifluorescence signal from excised brains and spinal cords. *Symbols* indicate statistical significance (#*p* < 0.05, **p < 0.005). Fold change compared to CFA controls is indicated in parentheses for the *in vivo* imaged mice. **(C)** Low and high power magnifications of cathepsin B expressing macrophages/microglia in a frozen section of an inflammatory lesion in the dorsal sacral spinal cord of a perfused EAE mouse injected with CB680 24 h previously. *Green* (anti-F4/80); *red* (CB680); *blue* (DAPI).

We next examined the potential of assessing protease activity as a biomarker of EAE severity by imaging mice at peak of acute disease with protease-activatable NIR fluorescence imaging agents: Cat B 680 FAST (CB680) for cathepsin B activity, ProSense 750EX (PS750) for pan-cathepsin activity (Cat B, L, S, K, V), MMPSense 750 FAST (MMP750) for pan-matrix metalloprotease activity (MMPs 2, 7, 9, 12, 13 and others), and ReninSense 680 FAST (RS680) for renin activity. The average clinical score of all PLP_139-151_-immunized mice for this study was 1.45 on day 14, with disease incidence of 53%. Asymptomatic mice were excluded from the study, and the overtly diseased animals showed an average clinical score of 2.75. Control mice were injected intravenously with agents 14 days after immunization with CFA; PLP_139-151_-immunized mice were injected with agents on the first day that they achieved a clinical score of 2 or 3 (days 13–14). Mice were imaged and analyzed as described in the study shown in Figures 2 and 3, and the data show the fluorescence (pmol) in brain and spine and the fold change as compared to control mice (Figure 4A). In addition, isolated brain and spinal cord tissue fluorescences (counts/energy) were assessed by epifluorescence imaging (Figure 4B).

CB680 imaging showed statistically significant elevations in the brains of EAE mice (3.6-fold increase relative to controls), and this was confirmed in *ex vivo* tissue imaging. Interestingly, neither RS680 nor MMP750 showed significant brain elevations, despite the association of MMPs and renin in EAE and the similar molecular weights as compared to CB680. In contrast, RS680 showed statistically significant elevations in the spinal cord, confirmed by *ex vivo* tissue assessment. CB680 also showed spinal cord elevation as confirmed both by *ex vivo* imaging and histological examination showing activated cathepsin B expression in F4/80^+^ macrophages/microglia in a spinal cord inflammatory lesion (Figure 4C), but the variability of the *in vivo* imaging in this particular study prevented attainment of statistical significance. One of the most interesting findings was that the pan-cathepsin imaging agent, PS750, did not show a signal within the brain and spinal cord despite its ability to detect cathepsin B in a manner similar to CB680. As the molecular weight of this agent is approximately 450,000 Da, it appears that this may have affected BBB passage.

### Imaging therapeutic efficacy of antigen-specific immune modulation in EAE mice

**Figure 5:**
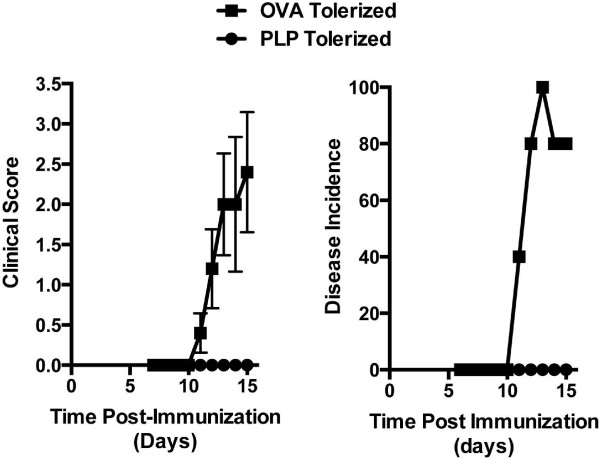
Clinical scores and disease incidence for control and PLP-tolerized mice **Clinical scores and disease incidence for control and PLP-tolerized mice.** Mice were injected with OVA_322-339_-conjugated or PLP_139-151_-conjugated PLGA nanoparticles 7 days prior to immunization with PLP_139-151_ in CFA (as described in Materials and Methods). Mean clinical scores (*left panel*) were assessed on days 7 through 15 following immunization, and disease incidence (*right panel*) was determined based on mice with a score of at least 1.

We have recently shown that intravenous injection of PLP_139-151_-coupled polystyrene or biodegradable poly(lactic-*co*-glycolic acid) beads (PLP-PSB or PLP-PLGA) can decrease EAE induction and severity induced by PLP_139-151_ immunization when using either prophylactic or therapeutic treatment regimens [20]. These particles are taken up via scavenger receptors on antigen-presenting cells to induce both pathogenic T cell hyporesponsiveness (anergy) and increased activity of regulatory T cells. We assessed the ability of CB680/AS750 imaging to detect tolerance-induced disease inhibition by treating mice with either OVA_323-339_-PLGA (OVA tolerized) or PLP_139-151_-PLGA (PLP tolerized) 7 days prior to immunizing mice with PLP_139-151_/CFA. The average clinical score of control OVA-tolerized mice was 2.4 by day 13, with disease incidence over 70% (Figure 5). In contrast, both average clinical score and disease incidence were significantly decreased in PLP-tolerized mice (clinical score 0; incidence 0%).

**Figure 6:**
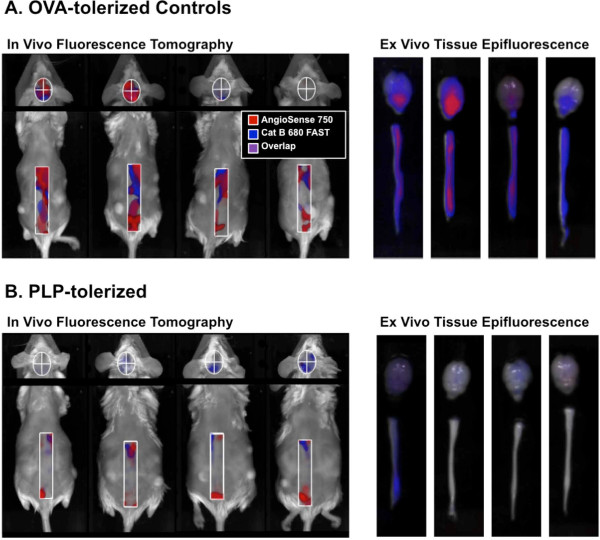
**NIR tomographic multiplex imaging AS750 and CB680 in control and PLP-tolerized mice. (A)** Tomographic images of vascular leak and cathepsin B activity in brain and lumbar/sacral spinal cord of control OVA-tolerized mice (*left panel*). Extraneous non-CNS signal was excluded for clarity. *Right panel* shows CB680/AS750 signal from excised brain and spinal cord tissue. **(B)** Tomographic images of vascular leak and cathepsin B activity in brain and lumbar/sacral spinal cord of control PLP-tolerized mice (*left panel*). Extraneous non-CNS signal was excluded for clarity. *Right panel* shows CB680/AS750 signal from excised brain and spinal cord tissue. Tomographic images from **A** and **B** are represented as fluorescence isosurfaces showing signal ≥65 nM (CB680 brain), ≥200 nM (AS7750 brain) and ≥100 nM (CB680/AS750 spine). Epifluorescence images are represented with the same monochrome color scales for both control and tolerized mouse tissues.

On day 13, all OVA- and PLP-tolerized mice were injected with a cocktail of CB680 and AS750 and imaged on day 14 (24 h later). Imaging results focused only on the signal within the spinal cord and brain regions for control OVA-tolerized (Figure 6A) and PLP-tolerized (Figure 6B) mice. Left panels show an overlay of AS750 and CB680 signal within the affected tissues in the living mice, with a clear difference apparent in the comparison of controls and tolerized mice. Very little signal is seen in the PLP-tolerized mice, whereas widespread regions of distinct and colocalized fluorescent signals are seen in the OVA-tolerized controls. As has been observed in previous studies, the brain signal in diseased mice tends to be much more variable than that in the spine regions; only half of the mice showed high brain signal. The right panels show the epifluorescence signal in the excised brain and spinal cord tissue, again represented as a two-color overlay of the two imaging agents. This *ex vivo* assessment correlates well with the non-invasive images acquired in the living mice.

**Figure 7:**
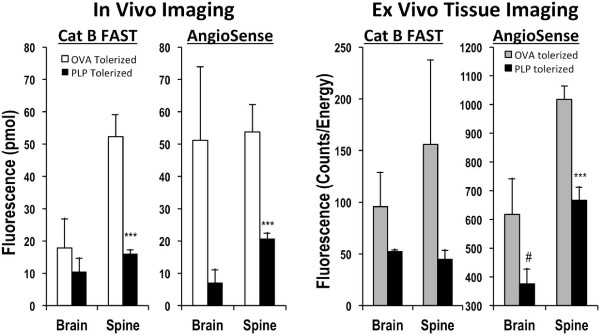
**Quantifying PLP-specific tolerance induction in EAE.** Fluorescence tomography and tissue epifluorescence were quantified for control and PLP-tolerized mice from the study represented in Figures 4 and 5. The *left panel* shows the quantification of *in vivo* tomographic fluorescence data sets. The *right panel* shows the quantification of epifluorescence signal from excised brains and spinal cords. *Symbols* indicate statistical significance (#*p* < 0.05, ***p < 0.0001).

Quantification of the two-channel fluorescent signal in EAE mice shows excellent detection of therapeutic efficacy of PLP tolerance induction when examining the spinal cord region (Figure 7) of the *in vivo* imaging data sets (*left panels*). Brain data, in contrast, showed the inherent variability of brain disease and a lack of correlation with clinical score. Thus, although there was a trend toward a decrease in brain signal with PLP-PLGA treatment, a much larger study would be required to assess this. The *ex vivo* brain and spinal cord tissue imaging by epifluorescence (*right panels*) confirmed *in vivo* observations and showed marginal statistical significance for AS750 brain signal.

## Discussion

Rodent models of EAE are used in research and drug development because of their similar pathology and/or pathogenesis to human MS and their ease of use and reproducibility. These models share a number of important morphologic and immunologic features with human MS, and they have contributed greatly to the overall knowledge of immune-mediated myelin destruction. Damage occurs in three phases; first some abnormal areas of tissue damage appear in the brain and spinal cord, followed later by leaks in the blood–brain barrier and then immune cell infiltration ultimately leading to demyelination [28]. EAE disease assessment relies heavily upon subjective clinical scoring of symptoms that capture little information regarding the molecular processes that drive these phases of disease. More detailed mechanistic information is typically obtained by histologic analyses of affected brain and spinal cord tissues.

New vascular and protease-activatable molecular imaging agents, as well as current improvements in imaging methods, provide an opportunity to gather non-invasive *in vivo* biological data regarding disease severity, progression and response to therapy. BBB compromise in EAE is well characterized [29], and AngioSense, a validated NIR vascular imaging agent [30,31], offers an opportunity for *in vivo* imaging of vascular leak into the CNS. In addition, as proteases play a central role in cancer and inflammatory disease processes, we anticipated that protease-activatable NIR imaging agents [32,33] could serve as a sensitive means for detecting neuroinflammation in EAE and changes with therapeutic intervention. Protease-activatable agents have been used to image protease increases in a number of disease states, including cancer [34,35], asthma [36,37], atherosclerosis [38,39] and arthritis [40,41]. However, it remained to be determined whether this collection of NIR imaging agents can pass the BBB and effectively detect disease within the CNS.

For the first time, we show the benefit of fluorescence molecular tomographic imaging in a mouse model of MS and demonstrate that tomography can not only provide images reflecting the disease severity, but also provide multiplex quantitative measurements (Figures 2, 3, 5 and 6). The first challenge in establishing a method for non-invasive EAE imaging of the brain and spinal cord was to determine whether our multiple potential agents, ranging in size from 33–450 kDa, could penetrate the BBB of mice. A number of preliminary studies with larger molecular weight AS750 (250 kDa) and activatable PS680 (450 kDa) revealed superior CNS accumulation by the somewhat smaller AS750 (data not shown), consistent with our findings in these studies. This led us to perform much of our early method validation with AS750, defining ROI shapes and sizes as well as optimal fluorescence thresholding approaches that yielded the technique described in Materials and Methods. All of our data to date have revealed considerable variability in brain disease (as also shown in the studies in Figures 4 and 6) and have further suggested that brain signal decreases dramatically in severely affected animals (clinical score of 4). Interestingly, PLP peptide-immunized animals with clinical scores of 0 or 1 often show as much brain fluorescence as mice with clinical scores of 2 or 3. This suggests that there can be some form of active disease occurring even in mice with no clinical symptoms, a finding that is not surprising as EAE clinical scores are mostly an assessment of spinal cord damage. Spine signal, on the other hand, generally correlates reasonably well with the clinical score but with more variability in mice with clinical scores of 4. Despite careful care of these animals, some of this decline in seriously diseased animals may be due to overall physiologic changes caused by dehydration and decreased circulation in these animals.

Optical tomographic imaging using NIR imaging agents in EAE has the potential for use in drug discovery research by virtue of deep tissue penetration, quantitative readout (pmol rather than light intensity) and the pairing with NIR imaging agents that detect the cellular participants in the underlying disease pathology. For the past 25 years, the Miller laboratory has explored specific T cell tolerance, induced by antigen-coupled splenocytes, as a means to both treat disease [42-45] and probe mechanisms of immunopathogenesis [46,47]. Recent advances in techniques for inducing T cell tolerance to specific neuroantigen epitopes have yielded an approach using antigen-coupled biodegradable PLGA nanoparticles to probe mechanisms of immune tolerance induction [20]. We applied our FMT imaging techniques with two of the best NIR agents identified from our validation studies, and were able to detect and quantify decreases in spinal cord vascular leak and cathepsin B activity in agreement with the effects on clinical scores (Figures 4, 5, and 6). Brain signal was variable, and half of the OVA-tolerized control mice showed little brain fluorescent signal with either agent. This highlights the variability of brain disease, but it also shows that vascular leak changes accompany cathepsin B changes as would be expected given that BBB compromise generally accompanies neuroinflammation. We were also able to confirm abnormal renin activity in EAE, in agreement with reports that a CNS renin-angiotensin system may be involved in the regulation of the disease [27]. Unfortunately, a similar agent for the detection of matrix metalloprotease activity (MMP750) was unable to detect changes in EAE despite the presence of MMP-secreting inflammatory cells. It is unclear whether unknown physical/chemical properties of this agent limited its CNS penetration or activity *in vivo*. Future studies will assess these agents, and additional NIR imaging agents, for their utility to detect disease as well as their sensitivity in detecting very early, subclinical disease.

Our studies have built upon advances in imaging technology to establish non-invasive optical tomographic imaging as a robust and accurate means of assessing BBB compromise and neuroinflammation in EAE *in vivo*. This technology offers a powerful tool for both basic neuroinflammation research and drug discovery and development by enabling the non-invasive monitoring of cellular processes that also drive human MS pathology. FMT thus should provide a rapid means for assessing and characterizing therapeutics for clinical evaluation.

## Abbreviations

BBB: Blood–brain barrier; CAT: Cathepsin; CFA: Complete Freund’s adjuvant; EAE: Experimental autoimmune encephalomyelitis; ECDI: 1-Ethyl-3-(3′-dimethylaminopropyl)carbodiimide; FMT: Fluorescence molecular tomographic imaging; MMP: Matrix metalloprotease; MRI: Magnetic resonance imaging; MS: Multiple sclerosis; NIR: Near infrared; OVA: Ovalbumin; PET: Positron emission tomography; PLGA: Poly(lactide-*co*-glycolic) acid; PLP: Proteolipid protein; ROI: Regions of interest; SPECT: Single photon emission computed tomography.

## Competing interests

Peterson and Vasquez are funded by PerkinElmer Inc., and this is clearly stated in the manuscript. The research documents the utility of PerkinElmer imaging agents and imaging technology in addressing specific biological questions in experimental autoimmune encephalomyelitis. The expectation is to further increase scientific interest in optical imaging in general and in PerkinElmer imaging agents and technology. As the technology (and imaging agents) have been around for several years (and have been published to some extent), the company expects no major financial gain as a result of publication beyond a modest increase in awareness in proportion to the robustness of the research. Northwestern University, Feinberg School of Medicine, employees have no financial stake in PerkinElmer Inc. The authors hold no patents relevant to the manuscript, and no patent applications are being submitted (or planned to be submitted). No reimbursement or funding relating to potential patents has been received. PerkinElmer currently holds patents for the technology used in the research.

## Authors’ contributions

JDP and SDM designed the research, analyzed data and wrote the paper; VLE, KOV, GEG and ZNH performed the experiments. All authors read and approved the final manuscript.
